# Medicinal signaling cells niche in stromal vascular fraction from lipoaspirate and microfragmented counterpart

**DOI:** 10.3325/cmj.2022.63.265

**Published:** 2022-06

**Authors:** Lucija Zenić, Denis Polančec, Damir Hudetz, Zeljko Jeleč, Eduard Rod, Dinko Vidović, Mario Starešinić, Srećko Sabalić, Trpimir Vrdoljak, Tadija Petrović, Fabijan Čukelj, Vilim Molnar, Martin Čemerin, Vid Matišić, Petar Brlek, Zrinka Djukić Koroljević, Igor Borić, Gordan Lauc, Dragan Primorac

**Affiliations:** 1 Department for Translational Medicine, Srebrnjak Children’s Hospital, Zagreb, Croatia; 2 St. Catherine Specialty Hospital, Zagreb, Croatia; 3 University Hospital Sveti Duh, Zagreb, Croatia; 4 School of Medicine, Josip Juraj Strossmayer University of Osijek, Osijek, Croatia; 5 Department of Nursing, University North, Varaždin, Croatia; 6 University Hospital Sestre Milosrdnice, Clinic for Traumatology, Zagreb, Croatia;; 7 School of Dental Medicine, University of Zagreb, Zagreb, Croatia; 8 Department of Traumatology, Merkur University Hospital, Zagreb, Croatia; 9 University of Zagreb Medical School, Zagreb, Croatia; 10 Medical School, University of Split, Split, Croatia; 11 Medical School, University of Rijeka, Rijeka, Croatia; 12 Medical School, University of Mostar, Mostar, Bosnia and Herzegovina; 13 University Department of Health Studies, University of Split, Split, Croatia; 14 Genos Glycoscience Research Laboratory, Zagreb, Croatia; 15 Faculty of Pharmacy and Biochemistry, University of Zagreb, Zagreb, Croatia; 16 Eberly College of Science, The Pennsylvania State University, University Park, State College, PA; 17 The Henry C. Lee College of Criminal Justice and Forensic Sciences, University of New Haven, West Haven, CT,USA; 18 Faculty of Dental Medicine and Health, Josip Juraj Strossmayer University of Osijek, Osijek, Croatia; 19 Medical School REGIOMED, Coburg, Germany

## Abstract

**Aim:**

To expand our previous findings by increasing the number of patients in a study characterizing medicinal signaling cells (MSC) of stromal vascular fraction from lipoaspirate (SVF-LA) and from microfragmented lipoaspirate (SVF-MLA) applied for the treatment of osteoarthritis (OA).

**Methods:**

Twenty OA patients, including 8 new patients, acquiring autologous microfragmented adipose tissue were enrolled. In-parallel immunophenotyping of SVF-LA and SVF-MLA was performed. The samples were incubated in a DuraClone SC prototype tube targeting the CD31, CD34, CD45, CD73, CD90, CD105, and CD146 surface markers, stained with the DRAQ7 cell nuclear dye and Live/Dead Yellow Fixable Stain, and analyzed by flow cytometry.

**Results:**

The population phenotypes in SVF-LA and SVF-MLA samples included CD31^+^CD34^+^CD73^±^CD90^±^CD105^±^CD146^±^ endothelial progenitors (EP), CD31^+^CD34^-^CD73^±^CD90^±^CD105^-^CD146^±^ mature endothelial cells, CD31^-^CD34^-^CD73^±^CD90^+^CD105^-^CD146^+^ pericytes, CD31^-^CD34^+^CD73^±^CD90^+^CD105^-^CD146^+^ transitional pericytes, and CD31^-^CD34^+^CD73^high^CD90^+^CD105^-^CD146^-^ supra-adventitial-adipose stromal cells. Compared with the autologous SVF-LA samples, the prevailing cell type in SVF-MLA were EP, which outnumbered leukocytes and supra-adventitial-adipose stromal cells (SA-ASC). The ratio of progenitor cells in SVF-MLA samples differed between female and male patients, showing a higher EP-pericyte and pericyte-SA-ASC ratio in men.

**Conclusion:**

Our results, hallmarked by EP-enriched anti-inflammatory features and indicating a possible sex-specific impact, contribute to defining the cellular composition of the clinically applied MSC serving as a regenerative cell therapy in OA.

Mesenchymal stromal/stem cells (MSC) are well known for their capability of differentiating into mesenchymal cell types. Caplan has recently suggested that they are renamed into medicinal signaling cells with the same acronym (1). The name ought to be adapted as our knowledge of the biological concept has expanded: during tissue regeneration, MSC perform their function via signaling rather by than differentiating – as they do under cell culture conditions (2-4). MSC comprise a heterogeneous population of stromal and stem cells with additional immunosuppressive and trophic properties, which upon injury or inflammation modulate the local environment by secreting numerous anti-apoptotic, anti-scaring, angiogenic, and mitotic factors (1,5). This paradigm for tissue regeneration has been brought up by the studies of MSC in osteoarthritis (OA), the most common joint disorder (6). MSC from adipose tissue are now widely investigated as a novel therapeutic method in the treatment of OA (7).

Although 20 years have passed since the first characterization of the multipotent MSC from adult adipose tissue, ie lipoaspirate (3), a complete characterization of this heterogeneous cell type remains elusive. This tremendous discovery opened up unprecedented possibilities in clinical application, however, undefined cellular heterogeneity and non-standardized protocols represent the main obstacle to the MSC usage in regenerative medicine. Together with other cell types, MSC are found in the stromal vascular fraction (SVF), which is obtained from adipose tissue upon collagenase treatment. Besides being a fruitful source of MSC, adipose tissue seems to potentiate the MSC-mediated tissue regeneration if it previously undergoes microfragmentation. The secretome of microfragmented adipose tissue more abundantly harbors cytokines and angiogenic factors, accompanied by immunomodulation, angiogenesis, and tissue reparation benefits (8). In this new light of paracrine activity of microfragmented adipose tissue, the Lipogems® device brings innovative technology for processing autologous adipose tissue, producing small intact clusters of perivascular microfragments with a high therapeutic potential (9,10). In a prospective non-randomized study, an intra-articular injection of such a product led to a successful outcome, as revealed by an increased glycosaminoglycan content in the hyaline cartilage of the knee joint (11,12). Several studies of knee OA treatment demonstrated the efficacy of adipose MSC in tissue repair, and even a low-dose MSC application yielded significant functional improvement with pain relief (13-15).

Although clinical implementation of microfragmented adipose tissue has brought an impressive step forward in orthopedics, standardization of clinical application requires a better understanding of MSC heterogeneity and the cellular subset characterization. The flow cytometry analysis of human lipoaspirate has shown that a heterogeneous SVF mixture contains endothelial progenitor (EP) cells (CD31^+^CD34^+^CD146^+^), endothelial mature (EM) cells (CD31^+^CD34^-^CD146^±^), pericytes (CD31^-^CD34^-^CD146^+^), supra-adventitial-adipose stromal cells (SA-ASC) (CD31^-^CD34^+^CD146^-^), and transitional pericytes (TP) (CD31^-^CD34^+^CD146^+^), with differential expression of the CD73, CD90, and CD105 mesenchymal markers (16,17). The aim of this study was to expand the number of patients from our previous immunophenotyping analysis of SVF from lipoaspirate (SVF-LA) or microfragmented lipoaspirate (SVF-MLA) by means of polychromatic flow cytometry (18). Since SVF-MLA is used therapeutically in OA patients, the results contribute to the biological understanding of the cartilage regeneration.

## MATERIAL AND METHODS

### Patients

We enrolled 8 new OA patients (4 women and 4 men) in addition to the previous 12 patients (6 women and 6 men) (all aged 30-85) undergoing an intra-articular knee injection of autologous SVF-MLA in St. Catherine Specialty Hospital (Zagreb, Croatia) as previously described (11). The study was approved by the St. Catherine Specialty Hospital Institutional Review Board (EP 001/2016) and the Srebrnjak Children’s hospital Research Ethics Committee (11/2017). The SVF-LA and SVF-MLA patient samples transported to the Srebrnjak Children's Hospital (Zagreb, Croatia) were stored overnight at room temperature (RT) protected from light before further processing (19,20).

### SVF isolation

The SVF was obtained by treating the samples with 1% collagenase type I in D-MEM medium (both from Sigma-Aldrich, Saint Louis, MO, USA) in a shaking bath at 37 °C for 45 minutes, accompanied by a 1:2 dilution with 2% fetal bovine serum (Biosera, Nuaille, France) in the D-MEM medium (Sigma-Aldrich) for stopping the reaction. Filtered through a 100 μm-cell strainer (BD Falcon, Corning, NY, USA), the samples were centrifuged (300 g for 10 min at RT) and the cell pellet was resuspended in 1 mL of the VersaLyse solution (Beckman Coulter, Miami, FL,USA) for a 10-minute incubation. After another filtration through a 40-μm cell strainer (BD Falcon, Corning), the samples were centrifuged (300 g for 10 min at RT), and the cell pellet was resuspended in the D-MEM medium (Sigma-Aldrich). The Sysmex XT1800 hematology analyzer (Sysmex, Kobe, Japan) was used to count the cells.

## Flow cytometry

The Duraclone SC dry reagent prototype tube (kindly provided by Beckman Coulter) was used for staining of the MSC subpopulation cell surface markers: CD31, CD34, CD45, CD73, CD90, CD105, CD146, labeled with PB, ECD, APC-AF750, PE, FITC, CD45-PC7, PC5.5 fluorochromes, respectively, together with Live/Dead Yellow Fixable Stain (Thermo Fisher, Waltham, MA,USA). The samples were incubated for 20 min at RT protected from light followed by fixation with 2% paraformaldehyde (Electron Microscopy Sciences, Hatfield, PA, USA) in PBS (Sigma-Aldrich). The samples were washed and permeabilized with PermWash (BD Biosciences, San Jose, CA, USA), and the cell nuclei were stained with the DRAQ7 dye (BioStatus, Shepshed, Leicestershire, UK). The FlowLogic software (Inivai Technologies, Mentone, Australia) and the Kaluza software (Beckman Coulter) were used to analyze the FCS data files.

### Statistical analysis

The normality of distribution was tested with the D'Agostino-Pearson test. The paired *t* test or unpaired *t* test, or Wilcoxon test or Mann-Whitney test were used to test the differences between the groups. Our previously published data sets were also included in the statistical analysis (n = 12) together with the new unpublished data obtained from additional patients (n = 8). A *P* value <0.05 was considered statistically significant. The analysis was performed with GraphPad Prism 9.2 for Windows (GraphPad Software, Inc., San Diego, CA, USA).

## RESULTS

### Polychromatic flow cytometry immunophenotyping of SVF-LA and SVF-MLA samples

**Figure 1:**
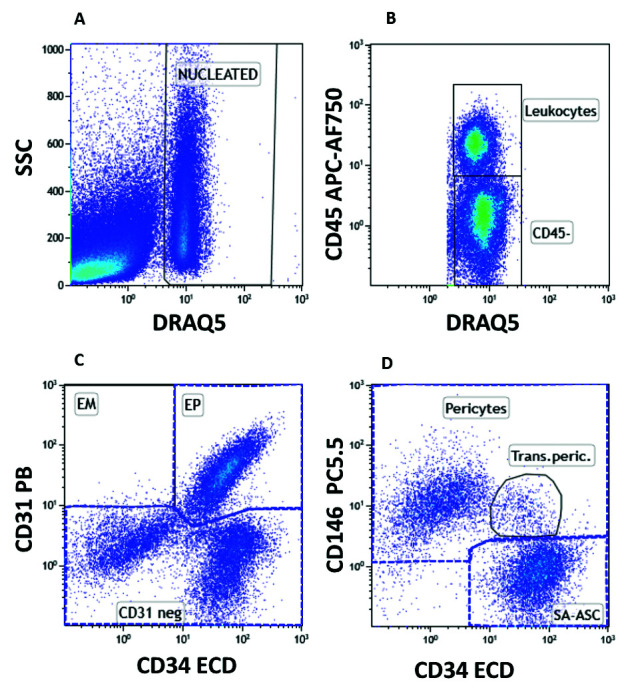
The gating procedure for flow cytometry analysis of the cellular content in the stromal vascular fraction derived from lipoaspirate or microfragmented lipoaspirate counterpart. Single live cells (data not shown) were used to analyze nucleated cells using the DRAQ7 dye and side scatter (SSC) (**A**) and the CD45^+^ leukocyte and CD45^−^ cell populations (**B**). Live nucleated CD45^−^ cells were defined with the CD31 and CD34 lineage markers and phenotyped as CD31^+^CD34^−^ endothelial mature (EM), CD31^+^CD34^+^ endothelial progenitor (EP), and CD31^−^ negative non-endothelial cells (**C**). The latter population was, in combination with the CD146 marker, further phenotyped as pericytes, transitional pericytes (TP), and supra-adventitial-adipose stromal cells (SA-ASC) (**D**).

Flow cytometry analysis of the SVF-LA or SVF-MLA counterpart samples was undertaken with the gating procedure (Figure 1). The singlet events chosen based on forward scatter time of flight and forward scatter area as well as the Live/Dead Yellow staining (not shown) were used for the DRAQ7 staining selection (Figure 1A). For the flow cytometry analysis, DNA-binding dye was used to select only nucleated cells and avoid abundant interference with oil drops, erythrocytes, and debris. Based on the CD45 expression, live nucleated cells were separated into the CD45^-^ non-leukocyte and CD45^+^ leukocyte fraction (Figure 1B). Using the CD31, CD34 and CD146 markers, in the non-leukocyte fraction we determined CD31^+^CD34^−^ EM, CD31^+^CD34^+^ EP, and CD31^−^ negative non-endothelial populations (Figure 1C), pericytes, TP, and SA-ASC (Figure 1D).

### SVF-LA and SVF-MLA significantly differed in cell content

**Figure 2:**
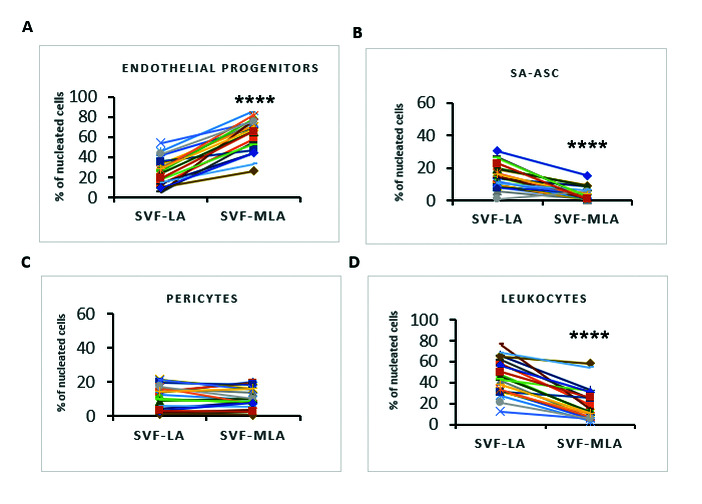
Differences in the four cell subpopulations before and after microfragmentation of lipoaspirate. The cell content of the stromal vascular fraction from lipoaspirate (SVF-LA) or microfragmented lipoaspirate (SVF-MLA) was calculated for endothelial progenitors (**A**), SA-ASC (**B**), pericytes, (**C**) and leukocytes (**D**) for each patient shown as a line. Statistical analysis was performed by using a paired *t* test (**A**, **B**, **D**) or Wilcoxon test (**C**). *P* value: (****) *P* < 0.0001; n = 20.

The heterogeneous cell content of SVF-MLA used for clinical application was analyzed for the relative amount of the main populations and compared with the SVF-LA counterpart (Figure 2). In SVF-MLA, the percentage of EP was significantly higher (Figure 2A), while the percentage of SA-ASC or leukocytes (Figure 2B and 2D) was significantly lower, in respect to the total nucleated cells. Interestingly, the percentage of pericytes was patient-dependent and did not differ between SVF-LA and SVF-MLA (Figure 2C). As previously shown, EM and TP comprised a small portion of the total nucleated cells (below 2%), which is why they were not included in the statistical analysis. These results supported our previous findings of the enrichment of the EP compartment in the clinically applied microfragmented SVF.

**Figure 3:**
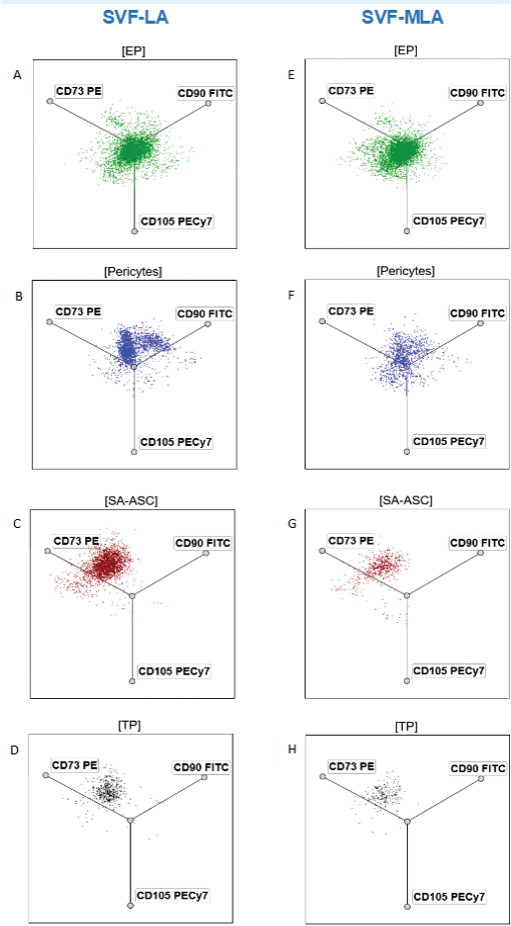
Expression analysis of the mesenchymal stem/stromal cell-characteristic markers on the progenitor cells of the stromal vascular fraction from lipoaspirate (SVF-LA) (left panels) or microfragmented lipoaspirate (SVF-MLA) (right panels). Expression of the CD73, CD90, CD105, and CD146 markers on endothelial progenitors (EP) (**A, E**), pericytes (**B, F**), supra adventitial-adipose stromal cells (SA-ASC) (**C, G**), and transitional pericytes (TP) (**D, H**). Data are expressed as radar plots of one patient with a more pronounced TP phenotype.

For a deeper insight into the heterogeneous MSC subpopulations, we compared the expression of the MSC-characteristic markers on the progenitor cells between the SVF-LA and SVF-MLA samples (Figure 3). The expression of CD73, CD90, CD105, and CD146 on EP (Figure 3A, 3E), pericytes (Figure 3B, 3F), SA-ASC (Figure 3C, 3G), and TP (Figure 3D, 3H) identified them as CD31^+^CD34^+^CD73^±^CD90^±^CD105^±^CD146^±^ EP, CD31^−^CD34^−^CD73^±^CD90^+^CD105^−^CD146^+^ pericytes, CD31^−^CD34^+^CD73^high^CD90^+^CD105^−^CD146^−^ SA-ASC, and CD31^−^CD34^+^CD73^±^CD90^+^CD105^−^CD146^+^ TP, respectively. The phenotype of EM was determined as CD31^+^CD34^−^CD73^±^CD90^±^CD105^−^CD146^±^ (not shown).

### Sex-related differences in the stromal progenitor cell ratios

**Figure 4:**
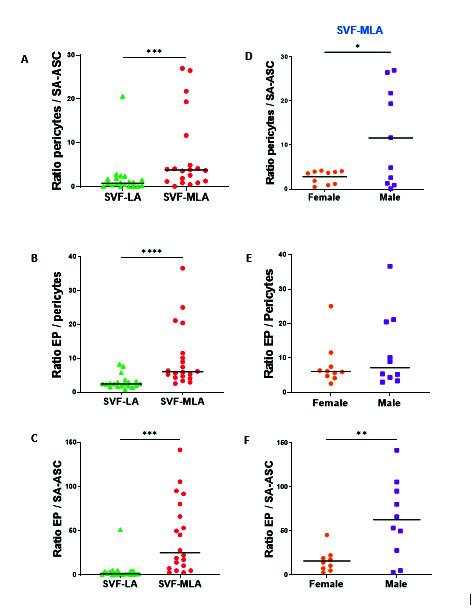
Ratio analysis of the progenitor cells in the stromal vascular fraction from lipoaspirate (SVF-LA) or microfragmented lipoaspirate (SVF-MLA). The pericyte/SA-ASC ratio (**A**), the EP/pericyte ratio (**B**), and the EP/SA-ASC ratio (**C**) were calculated from the percentage of nucleated cells for each cell population (Figure 2). The data are expressed as symbols representing each patient with the group median, and the statistical analysis was performed with the Wilcoxon test (A-C). *P* values: (***) *P* < 0.001, (****) *P* < 0.0001; n = 20. Differences between female and male patients in the pericyte/SA-ASC ratio (**D**), the EP/pericyte ratio (**E**), and the EP/SA-ASC ratio (**F**) in SVF-MLA samples. The data are expressed as symbols representing each female patient (n = 10) or male patient (n = 10) with the group mean (D, F) or median (**E**). Statistical analysis was performed with the unpaired *t* test (D, F) or Mann-Whitney test (**E**). *P* values: (*) *P* < 0.05, (**) *P* < 0.01.

We were next interested in the ratio of the progenitor cells, which might be important for their *in vivo* interaction and local modulation upon an intra-articular injection. SVF-MLA samples had significantly higher ratios of pericytes/SA-ASC, EP/pericytes, and EP/SA-ASC than SVF-LA samples (Figure 4A-C). As we observed earlier, male samples showed significantly higher values than female samples, except for the EP/pericytes ratio (Figure 4D-F). Notably, one female patient's data in Figure 3F was identified by the ROUT method as an outlier and was excluded from the statistical analysis.

## DISCUSSION

This study strengthens our published results on the immunophenotyping of the SVF cell components from MLA previously proven to improve the outcome in patients with the late-stage knee OA (11,12). We described five CD45^-^ subpopulations of SVF-LA and SVF-MLA of the following phenotypes: CD31^+^CD34^+^CD73^±^CD90^±^CD105^±^CD146^±^ EP cells, CD31^+^CD34^-^CD73^±^CD90^±^CD105^-^CD146^±^ EM cells, CD31^-^CD34^-^CD73^±^CD90^+^CD105^-^CD146^+^ pericytes, CD31^-^CD34^+^CD73^±^CD90^+^CD105^-^CD146^+^ TP, and CD31^-^CD34^+^CD73^high^CD90^+^CD105^-^CD146^-^ SA-ASC. The expression pattern of the MSC markers on TP resembles that of SA-ASC, to which they develop from pericytes (16).

Previous attempts focusing on the *in situ* origin and immunophenotyping of adipose tissue MSC have been hindered by the fact they cannot be unambiguously identified by any current marker (21). In addition, many studies assessed the expression of a single marker, such as the percentage of the CD34-positive or CD146-positive population within the nucleated CD45- cells. However, these are not population-specific markers but are markers shared by different subpopulations, and the cellular composition of SVF with the specific ratios remained undefined. Instead, a combination of different MSC-characteristic markers in a polychromatic flow cytometry analysis, which we applied, provides a better insight. A similar analytical approach to ours was applied by Vezzani (8) and Zimmerlin (17).

Although SA-ASC and pericytes might not share a common origin (22), they are widely presumed to be the *in vivo* progenitors of MSC (23,24). However, we observed EP to be the most abundant progenitor cell type in SVF-MLA, which accounted for the most fruitful enrichment after microftagmentation. Interestingly, the percentage of pericytes did not depend on lipoaspirate processing and was unchanged after microfragmentation. Applied as an autologous cell therapy in patients with late-stage knee OA, SVF-MLA improved the outcome even in patients initially considered for a knee replacement surgery (12). The notion that the clinically applied SVF-MLA was abundantly enriched in EP cells has implications for their role in the MLA-mediated cartilage improvement. EP are bone marrow-descendent unipotent progenitor cells capable of differentiating to endothelial cells, as well as to contribute in a paracrine manner to the process of vascularization necessary for tissue regeneration (25,26). Their pericyte origin has also been proposed (17). Co-cultures of EP and MSC have a synergistic effect in angiogenesis and bone regeneration (27,28) as well as in differentiation commitment (29-31). Experiments using adipose tissue MSC and bone marrow EP have shown that these co-cultures generated more bone and cartilage (32), upregulated osteogenesis-related and angiogenesis-related gene expression (33), and in animal models accelerated bone defect repair (33). Extensive *in vitro* and *in vivo* research has thus highlighted vasculogenic and proangiogenic effects resulting from the MSC-EP cross-talk (34-36). These studies have identified several ways of MSC-EP communication, including a direct contact, and interactions via soluble factors or extracellular vesicles (37-39). Therefore, it is reasonable to suspect that MSC in interactions with EP exert their medicinal action and perhaps mediate the observed cartilage regeneration after injection of microfragmented SVF. In line with the previous reports (10), we observed a decrease in CD45^+^ leukocytes in SVF-MLA. MSC are known for their anti-inflammatory action, and a reduction of proinflammatory elements in the microfragmented mixture most likely contributes to the MSC effects at the site of injury.

Interestingly, we found that women and men had different pericyte/SA-ASC and EP/SA-ASC ratios, which implicates the influence of sex hormones. Similar to our finding, Vezzani et al (8) also documented a pericyte domination over SA-ASC that resulted from microfragmentation. Their study involved only women, and these results were similar to our data from female patients. In our study, EP and pericytes as the two most dominant populations of the microfragmented product did not show sex-related variations. Women and men differ in fat composition and stores, and adipose tissue-derived stem cells from men are characterized by a faster proliferation or stronger osteogenesis than those from women (40-42). Women with a higher plasma estrogen concentration have higher levels of circulating EP, which can, together with MSC, in a paracrine manner mediate the estrogen effects (43). However, although the sex-related discrepancies that we observed appear to be very significant, the study was performed on a small number of patients and the results are yet to be confirmed in a broader study with a larger number of patients. Additionally, the association with the clinical outcome after intra-articular knee injection of autologous SVF-MLA in men and women needs to be assessed.

In conclusion, since SVF-MLA is used therapeutically in OA patients, the results of this study may contribute to the biological understanding of the cartilage regeneration. Here, we successfully used the Duraclone SC Mesenchymal tubes for the SVF immunophenotyping and confirmed a marked increase in EP in microfragmented adipose tissue. The fact that SVF-MLA was enriched in EP cell population, with a concomitant reduction of leukocytes, might help in explaining its anti-inflammatory and regenerative properties in OA joint healing.
